# Traditional and Molecular Techniques for the Study of Emerging Bacterial Diseases: One Laboratory’s Perspective

**DOI:** 10.3201/eid0802.010141

**Published:** 2002-02

**Authors:** Pierre Houpikian, Didier Raoult

**Affiliations:** Unité des Rickettsies, Faculté de Médecine de Marseille, Marseille, France

**Keywords:** emerging bacteria, unrecognized pathogens, bacterial detection, bacterial identification, histologic stain, broad-range PCR, 16S rDNA, culture, animal model, complete nucleotide sequence

## Abstract

Identification of emerging bacterial pathogens generally results from a chain of events involving microscopy, serology, molecular tools, and culture. Because of the spectacular molecular techniques developed in the last decades, some authors think that these techniques will shortly supplant culture. The key steps that led to the discovery of emerging bacteria have been reviewed to determine the real contribution of each technique. Historically, microscopy has played a major role. Serology provided indirect evidence for causality. Isolation and culture were crucial, as all emerging bacteria have been grown on artificial media or cell lines or at least propagated in animals. With the use of broad-range polymerase chain reaction, some bacteria have been identified or detected in new clinical syndromes. Culture has irreplaceable advantages for studying emerging bacterial diseases, as it allows antigenic studies, antibiotic susceptibility testing, experimental models, and genetic studies to be carried out, and remains the ultimate goal of pathogen identification.

In the last 20 years, advances in knowledge have resulted in a broad expansion of the spectrum of microorganisms regarded as human pathogens. Most advances have evolved in a series of small steps based on several techniques that have been used successively by different investigators who faced clinically suspect diseases. These include the traditional techniques of microscopy, serology, and culture, as well as more recent molecular tools (Figure 1). In addition to aiding in discovering new pathogens, these techniques also contributed to studies of the epidemiology, pathophysiology, and treatment response of the newly recognized diseases, providing further evidence for causal relationships between disease and organism (*1*). As a diagnostic and research laboratory specializing in fastidious, intracellular bacteria, we have been particularly interested in assessing the specific role played by culture in identifying emerging pathogens. Historical examples, such as Lyme or Legionnaires’ diseases, and recent successes, such as culture of the Whipple bacillus, support the effectiveness of this technique (*2*). Moreover, culture provided the basis of other supplemental tools to elucidate the causes of microbial disease and to study the clinical and biological features of emerging bacterial diseases. These tools are not only antigenic and serologic assays but also in vitro and in vivo disease models for pathophysiologic studies and antimicrobial susceptibility testing, plus extensive genetic sequencing. The isolation of emerging pathogens serves, therefore, not only as a means for diagnosis but also as a route to enhance understanding of the diversity and epidemiology of emerging bacteria and the infections they cause.

**Figure 1:**
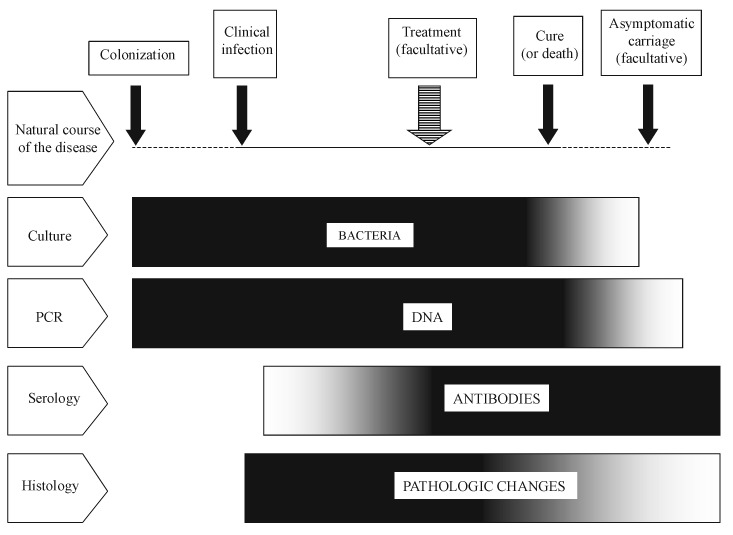
Diagram describing the respective places of culture-, polymerase chain reaction-, serology- and histology-based approaches for the diagnosis of acute bacterial infections, according to the natural course of the disease. Isolation and culture are possible as long as live bacteria are present in tissues, i.e., from the colonization to the treatment or to the end of the clinical manifestations (or shortly earlier). Bacterial DNA can be detected during the same period and also as far as dead microorganisms remain in tissues. Specific antibodies appear during the clinical course of the disease and persist generally for months or years. Pathologic changes can be observed soon after the contamination and, in an acute infection, will decline rapidly after elimination of the bacteria.

Despite these unique advantages, however, culture has been challenged by the recent development of genotype-based methods such as broad-range polymerase chain reaction (PCR) (*3*). Because culture as a tool is still threatened by the possible existence of uncultivatable organisms, several authors have emphasized the critical role that molecular, culture-independent techniques could play in further investigations of emerging infectious diseases, affirming that a reassessment of Koch’s postulates for disease causation was required (*4*). What actually are the respective roles of these two techniques? Should we consider that broad-range PCR has made culture and traditional techniques obsolete, or is it only a step among others in the sequence of events leading to isolation of a new microorganism? To answer these questions, we examined the key steps that led to identification of most bacterial diseases that have been discovered during the last 20 years. Table 1 presents the main biological evidence that allowed merging bacteria to be recognized and disease causation to be demonstrated**.** We examined the contribution of traditional and molecular techniques to understand their respective roles, and we emphasize the specific advantages of culture.

**Table 1:** Key steps that led to identification and demonstration of disease causation for emerging bacteria^a^

Group	Species	Clinical picture	Histologic detection	Serology	Molecular Detection (gene**)**	Culture system	Year of culture	Ref.
Alpha1 Proteobacteria								
	*Ehrlichia chaffeensis*	Fever, cytopenia	Smear	Antibodies to *Ehrlichia canis****]***	16S rRNA	Cell line (DH82)	1991	*5*
	*E. ewingii*	Fever, cytopenia	Smear	Western blot	16S rRNA	Cell line	1971	*6*
	Human granulocytic *Ehrlichia*	Fever, cytopenia	Smear	Antibodies to *E. phagocytophila, E. equis*	16S rRNA	Cell line (HeLa)	1996	*7*
	Rickettsia felis	Fever			*gltA*	Cell line (XTC-2)	2000	*8*
	*R. japonica* ***]***	Spotted fever		Antibodies to Spotted fever group rickettsiae		Cell line (Vero)	1989	*9*
	*R. mongolotimonae*	Febrile rash		Antibodies to Spotted fever group rickettsiae	*rOmpA*	Embryonated egg, guinea pig	1991	*10*
	*R. slovaca*	Fever, eschar, lymphadenitis		Specific antibodies	*rOmpA*	Cell line	1968	*11*
Alpha2 Proteobacteriae								
	*Afipia broomae*	Wrist abscess				Axenic (specific)	1981	*12*
	*A. clevelandensis*	Osteitis				Axenic (specific)	1988	*12*
	*Bartonella elizabethae*	Endocarditis				Axenic (nonspecific)	1993	*13*
	*B. grahamii*	Neuro-retinitis		Antibodies to *B. henselae*	16S rRNA	Axenic (nonspecific)	1995	*14*
	*B. henselae*	Fever, cat scratch disease, bacillary angiomatosis	Tissue section	Specific antibodies	16S rRNA	Axenic (nonspecific)	1990	*15* *,* *16*
Beta Proteobacteriae								
	*Bordetella trematum*	Chronic otitis				Axenic (nonspecific)	1996	*17*
	*Neisseria weaveri*	Infected wound				Axenic (nonspecific)	1993	*18*
Spirochetae								
	*Borrelia burgdorferi *sensu stricto*, B. afzelii, B. garinii*	Erythema chronicum migrans, acrodermatitis chronica atrophicans, Lyme arthritis, neuro-borreliosis		Specific antibodies		Axenic (specific)	1981	*19*
	*B. duttonii*	Relapsing fever	Smear	Specific antibodies		Axenic (specific), animal model	1999	*20*
	*B. recurrentis*	Relapsing fever	Smear			Axenic (specific)	1994	*21*
Delta-Xi Proteobacteriae								
	*Campylobacter coli, C. jejuni*	Febrile diarrhea		Specific antibodies		Axenic (nonspecific)	1977	*22*
	*Helicobacter cinaedi, H. fennelliae*	Rectitis				Axenic (nonspecific)	1984	*23*
	*H. heilmanii*	Chronic gastritis	Tissue section			Mouse	1989	*24*
	*H. pylori*	Gastritis, gastroduodenal ulcer	Tissue section	Specific antibodies		Axenic (nonspecific)	1982	*25*
Gamma Proteobacteriae								
	*Escherichia coli* O48:H21, O103:H2, O157:H7	Bloody diarrhea, HUS			*slt*	Axenic (nonspecific)	1982-1996	*26*
	*Haemophilus influenzae* biogroup *aegyptius*	Brazilian purpuric fever				Axenic (nonspecific)	1986	*27*
	*Legionella anisa*	Pneumonia, Pontiac fever		Specific antibodies		Axenic (specific)	1989	*28*
	*L. bozemanii*	Pneumonia	Smear	Specific antibodies		Axenic (specific)	1983	*29*
	*L. dumoffii*	Pneumonia	Smear	Specific antibodies		Axenic (specific)	1978	*29*
	*L. feeleii*	Pneumonia, Pontiac fever		Specific antibodies		Axenic (specific)	1986	*30*
	*L. micdadei*	Pneumonia		Specific antibodies		Embryonated egg, guinea pig	1979	*29*
	*L. oakridgensis*	Pneumonia	Smear	Specific antibodies		Axenic (specific)	1987	*29*
	*L. pneumophila*	Pneumonia	Tissue section	Specific antibodies		Embryonated egg, Guinea pig	1947	*31*
	*Legionella* like amoebal pathogen	Pneumonia		Specific antibodies		Amoeba	1991	*32*
	*Vibrio alginolyticus*	Conjunctivitis, wound infection				Axenic (nonspecific)	1977	*33*
	*V. cholerae* O:139	Diarrhea				Axenic (nonspecific)	1992	*33*
	*V. fluvialis*	Diarrhea				Axenic (nonspecific)	1980	*33*
	*V. furnissii*	Diarrhea				Axenic (nonspecific)	1983	*33*
	*V. metschnikovii*	Cholecystitis				Axenic (nonspecific)	1981	*33*
	*V. mimicus*	Diarrhea, otitis				Axenic (nonspecific)	1981	*33*
Mycobacteria								
	*Mycobacterium asiaticum*	Pneumopathy				Axenic (specific)	1983	*34*
	*M. celatum*	Pneumopathy				Axenic (specific)	1992	*34*
	*M. genavense*	Disseminated infection, lymphadenitis	Tissue section			Axenic (specific)	1992	*34*
	*M. malmoense*	Pneumopathy, lymphadenitis				Axenic (specific)	1977	*34*
	*M. simiae*	Pneumopathy, osteitis, kidney infection				Axenic (specific)	1984	*34*
Mycoplasmas								
	*M. fermentans*	Pneumopathy, nephritis	Tissue section		Insertion sequence-like	Axenic (specific)	1993	*35*
	*M. genitalium*	Urethritis	Smear		Adhesion protein	Axenic (specific), Animal model	1981	*35*
Gram-positive bacteria								
	*Tropheryma whipplei*	Whipple disease	Tissue section	Specific antibodies	16S rRNA	Cell line (HEL)	2000	*2* *,* *36*
	*Corynebacterium auris*	Acute otitis				Axenic (nonspecific)	1995	*37*
	*Staphylococcus lugdunensis, S. schleiferi*	Skin abscess, osteoarthritis				Axenic (nonspecific)	1988	*38*
	*Streptococcus iniae*	Meningitis, endocarditis, cellulitis				Axenic (nonspecific)	1995	*39*

^a^Histologic detection can be performed with morphologic techniques, in blood or tissue smears, or in tissue sections. Serologic assays can detect specific antibodies to the suspected agent or to a related organism in tissues or in biological fluids. The year of the first isolation and the culture system used are indicated. HUS = hemolytic uremic syndrome. HLE=human embryonic lung fibroblasts; ref = reference.

## Traditional Techniques Other Than Culture: Microscopy and Serology

### Optic Microscopy

#### Direct Detection in Smears

Historically, morphologic methods have played an important role in detecting new microorganisms, and they are still crucial for diagnosing infections caused by agents not routinely cultured, such as *Mycobacterium leprae* (*40*). Because microscopic examination of stained smears from biologic fluids or tissue imprints is usually rapid and easy, it has often been performed in patients who have an unexplained disease, although its interpretation is subjective and its sensitivity and specificity are generally low. The first evidence for the responsibility of *Ehrlichia* species in humans with an acute febrile illness was provided by examining blood smears stained with a Romanowsky stain, in which these as-yet-uncultivated organisms could be observed forming intracytoplasmic morulae within leukocytes (*6*,*7*). *Borrelia burgdorferi* were first observed in Giemsa-stained smears from midgut diverticula of ticks (*19*). Examination of smears can also be helpful when multiple organisms are cultured from a nonsterile site, as microbial culture alone, as well as molecular detection, cannot distinguish between colonization or asymptomatic shedding and tissue invasion: in such a situation, the morphology of the predominant organism visualized in the tissue sections can suggest the true causative agent (*40*).

#### Detection in Tissue Sections

Although individual bacteria generally are not detected in hematoxylin and eosin (H&E)-stained tissue sections, exceptions do exist. Clumps of finely particulate basophil material were seen in H&E-stained sections of bacillary angiomatosis and subsequently identified as *Bartonella* (*41*). In H&E-stained sections of gastric biopsy specimens that show acute gastritis, curved bacteria consistent with *Helicobacter pylori* may be seen in the layer of mucus on the crypt epithelium (*25*). Moreover, as histopathologic damage and causal microorganisms usually have a long-established association, microscopic examination of H&E-stained tissue sections during the course of an unexplained disease may lead to hypotheses about the nature of the etiologic agent (*40*).

**Figure 2:**
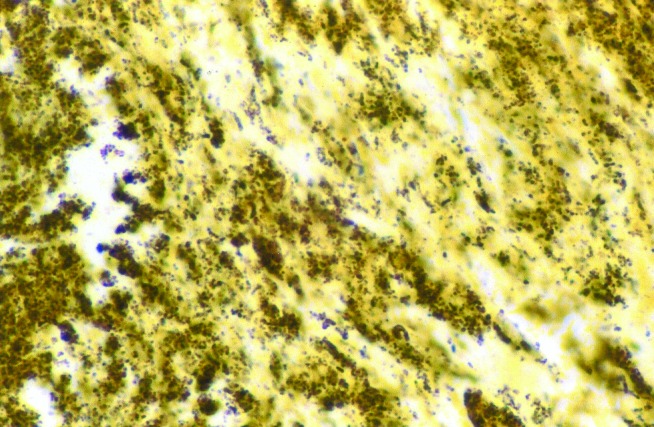
Demonstration of Bartonella henselae in cardiac valve of a patient with blood culture-negative endocarditis. The bacilli appear as black granulations (Warthin Starry, original magnification X250).

Gram stain has also proven useful to routinely diagnose *H. pylori* and *H. heilmanii* in the gastric mucosa of patients with gastritis, as well as that of *B. henselae* in cardiac valves (*10*,*24*,*25*). Silver impregnation is among the most useful methods for detecting bacteria, especially for that stained weakly with a tissue Gram stain. Thus, bacillary angiomatosis lesions were found to contain clusters of bacilli on Warthin-Starry staining 2 years before the etiologic role of *B. henselae* was elucidated. With the same stain, this bacterium was also detected in cardiac valves of patients with endocarditis (Figure 2) (*41*). The first observation of Whipple agent was reported in 1907 by George Whipple in silver-stained sections of a lymph node, although the author did not link this observation with the cause of the disease (*2*).

Special stains have also played a role in establishing the etiologic role of new bacteria. Gimenez’ and Pinkerton’s stains allowed the detection of rickettsial organisms in tissue sections from patients with acute febrile disease (*40*).New mycobacteria were initially detected by using Ziehl-Nielsen, Kinyoun, or auramine O stains. For example, in an HIV-infected boy, examination of a retroperitoneal lymph node showed granuloma with large numbers of intracellular acid-fast bacilli that were later characterized as a new *Mycobacterium* species, *M. genavense* (*34*). Morphologic techniques, indeed, do not allow specific identification of the detected organisms. Despite this limitation, the approach consisting of detecting infectious lesions and agents by using cytologic and histologic examination appeared to be sometimes more valuable than the cultural or molecular techniques (*40*).

### Electron Microscopy

Among morphologic techniques, transmission and scanning electron microscopy (EM) has substantial advantages resulting from its high flexibility and sensitivity (*42*). Negative staining is a rapid EM method that can be useful in patients with persisting or unexplained disease. Further, its specificity and sensitivity can be enhanced by using immuno-capture assay**.** Thus, in patients with chronic gastritis, EM provided the first detection of *H. pylori* in the gastric mucosa (*25*). EM can resolve details many hundreds of time smaller than can be seen through light microscopes, and resolution of major taxonomic features can help to characterize new microorganisms (*42*). Thus, the agent of Whipple disease was recognized as a bacillus through ultrastructural examination of the bacilli (*42*). Nevertheless, limitations of EM include its availability, cost, and need for experienced staff. EM requires knowledge of histology and ultrastructure of the tissue being examined and organisms likely to be encountered and is very time-consuming, since every specimen must be examined individually (*42*).

## Serology and Antigenic Detection

### Serology

By showing rising antibody titers or seroconversion, serology can provide indirect evidence for causal relationships between a disease and a newly identified bacterium. Conversely, in the absence of serologic evidence, the role of a cultured organism should be interpreted cautiously, as shown by the example of *Afipia felis,* which was first thought to be the cause of cat-scratch disease, but was finally identified as a water contaminant (*12*,*43*). Serology is also useful to assess the involvement in human diseases of microorganisms that had been initially recovered from the environment, such as novel *Legionella* species, or from animal hosts, as for the tick-associated bacteria *Borellia burgdorferi* or *Rickettsia slovaca* (*11*,*19*,*29*). Further, serology is a valuable tool for exploring the disease spectrum of a bacterium. Thus, serologic testing contributed to the recognition of *B. henselae* as the main agent of cat-scratch disease (*16*), as well as implicating *Campylobacter jejuni* as a possible cause of Guillain-Barré syndrome (*44*).

Moreover, the contribution of serologic studies to the identification of new bacterial pathogens should not be underrated. Serologic cross-reactions are common between members of the same bacterial genus, and antibodies specific to a bacterial species can suggest the role of a closely related, still unidentified organism. Thus, specific antibodies to *Ehrlichia canis*, *E. phagocytophila*, and *E.* *equis*, then known only as veterinary pathogens, were detected in patients and led to description of the agents of human ehrlichioses (*E. chaffeensis*, *E. ewingii*, and human granulocytic ehrlichiosis) (*5*–*7*). Involvement of *Bartonella grahamii* in neuroretinitis was first suggested by detection of specific antibodies to *B. henselae* in the patient’s blood (*14*). Reliable interpretation of such serologic cross-reactions, however, would not have been possible without considering other evidence, such as intraleukocytic morulae for ehrlichioses.

### Antigenic Detection

**Figure 3:**
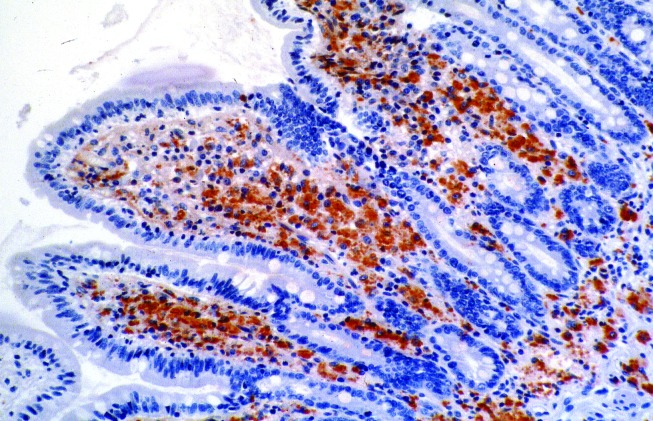
Demonstration of Tropheryma whipplei by immuno-histochemistry in the lamina propria of the villous tips. Bacilli are revealed in foamy macrophage cytoplasm as red-brown deposits (polyclonal rabbit.

Production of specific antibodies in experimental animal studies allowed immunochemical detection techniques to be developed. Direct immunofluorescence staining can be performed in smears in respiratory fluids of patients with pneumonia (*29*). Immunohistochemistry is useful for demonstrating disease causation, as it provides evidence for in situ association between microorganisms and histologic structures. With this technique, *Tropheryma whipplei* was detected in a patient’s mitral valve and later in intestinal mucosae (Figure 3) (*2*). Immunohistochemistry also suggested the role of *M. fermentans* in pulmonary infections (*35*). Immunologic techniques are dependent, however, on the availability of specific antibodies or antigens, which in most cases requires previous isolation of the agent; therefore, such techniques indirectly contribute to culture.

## Culture: A Traditional Technique of Expanding Potential

### Culture Media

#### Axenic Media

Broad-spectrum media allowed several previously unrecognized gram-positive bacteria, such as novel corynebacteria or *Staphylococcus* species, as well as novel beta-Proteobacteria, to be isolated, mainly from blood or pus of patients (*18*,*37*,*38*). The first isolation of *B. elizabethae, B. quintana*, and *B. henselae* was also achieved on blood agar (*15*). Use of *Campylobacter*-selective medium allowed novel *Campylobacter* and *Helicobacter* species to be grown from stools and rectal swabs, respectively (*23*), and provided further evidence for the association between *C. jejuni* infection and Guillain-Barré syndrome (*44*). For *Campylobacter* spp., selective, antibiotic-containing media could be satisfactorily replaced by nonselective blood agar, provided stool specimens had been filtered with a cellulose acetate membrane (*23*). Newly recognized serotypes of enterohemorrhagic *Escherichia coli* were isolated on MacConkey-sorbitol agar from stools or urine of patients with hemolytic-uremic syndrome (*26*). For *Vibrio cholerae* O:139 and most novel *Vibrio* species, the most convenient, highly selective medium was thiosulfate-citrate-bile salts sucrose agar (*33*).

The usefulness of broad-spectrum media should not obscure the fact that some emerging bacteria would not have been isolated without specific media. Buffered charcoal-yeast extract (BCYE) agar facilitated the recovery of most novel *Legionella* species, as well as *Afipia broomeae* and *A. clevelandensis,* from human respiratory sources (*12*,*29*) The first cultivation of *Borrelia burgdorferi* was achieved in 1981 in a modified Kelly medium (*19*). In 1994, 20 years after the first attempts, the Kelly growth medium itself allowed first cultivation of *B. recurrentis* from the blood of an Ethiopian patient with louse-borne relapsing fever, and *B. duttonii*, agent of East African tick-borne relapsing fever, was isolated for the first time in 1999 in BSK II medium (*21*). Generally, combining different types of medium, using both solid and liquid media, increases the effectiveness of culture, perhaps because of a preference of the bacterium for one type of medium over another or simply from the increased sensitivity obtained by culturing a large volume of specimen. For example, *B. elizabethae* and *B. henselae* were detected in BACTEC blood culture medium before inoculation in blood agar (*13*,*15*). Isolation of most novel *Mycobacterium* species required both solid- and liquid-specific media (*34*).

#### Living Systems

While more expensive and less easy to use than artificial media, animal models can provide certain advantages not available with artificial media. For example, until recently, inoculation to mice was the only means available to propagate *B.* *duttonii* (*21*). Today, animals are still necessary for isolating organisms such as *Treponema pallidum* or *Mycobacterium leprae*. Animal inoculation can help to reduce the contaminant flora. Thus, a combination of passage in guinea pigs and subsequent transfer into embryonated eggs was the key for isolating *L. pneumophila* from lung autopsy specimens (*31*). Embryonated eggs themselves have been recognized as a standard for rickettsial isolation, allowing, for example, the first isolation of Astrakhan fever rickettsia (*45*).

**Figure 4:**
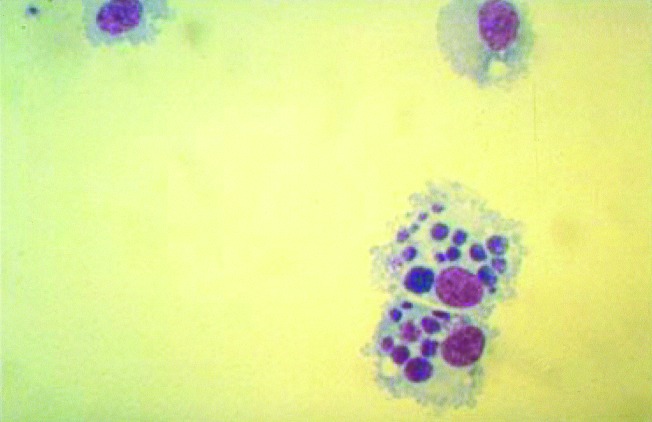
Canine monocytes (DH82) cultivated in vitro and heavily infected with Ehrlichia chaffeensis, as viewed by light microscopy after Giemsa staining. Typical ehrlichial inclusions (morulae) are observed within the cytoplasm of the infected cells (Giemsa, original magnification X600).

Cell culture is easy to use and may be very sensitive. Isolation of *T. whipplei* was obtained from valve and duodenal biopsy specimens by using human embryonic lung fibroblasts (HEL) (*2*,*36*). *Ehrlichia chaffeensis* and *R. japonica* were grown from blood samples of patients on canine macrophage cells (Figure 4) and African green monkey cells, respectively (*9*,*46*). Cultivation of facultative intracellular bacteria also was facilitated by cell culture. *L. pneumophila* has been isolated by using HEL cells while inoculated BCYE and agar plates remained sterile (*47*). With a bovine endothelial cell line, *B. quintana* was isolated for the first time from cutaneous biopsy material of a bacillary angiomatosis patient (*48*). Such enhanced sensitivity is a major advantage for an infection with low levels of bacteremia or when limited biopsy material is available (*49*). Indirectly, HEL cells also provided the first evidence for the role of a toxic factor in pseudomembranous colitis, which could be neutralized by clostridial antiserum. This observation led to the discovery of *Clostridium difficile* as the responsible agent (*50*).

The search for appropriate media that could allow the growth of still uncultivatable or unrecognized bacteria has led us to try coculture with nonmammalian cells. Cell lines from toads (XTC-2) have been used in our laboratory to grow *Rickettsia felis*, a flea-associated *Rickettsia* pathogenic for humans (*8*). Coculture with arthropod cells will probably enhance our ability to detect intracellular, arthropod-transmitted bacteria. For example, tick cells (IDE8) have been used to grow the agent of human granulocytic ehrlichiosis (*7*). Cocultivation of samples with free-living amebae has allowed recovery of otherwise uncultivatable microorganisms from patients and the environment. This technique provided evidence for the role of several *Legionella* or *Legionella*-like species and of *Parachlamydia acanthamoeba* as etiologic agents of community-acquired pneumonia (*51*).

### Other Critical Issues in Culture

In addition to the choice of an appropriate medium, the main critical issues in culturing concern inoculation of the specimen and incubation of the culture, both summarized in Table 2. Since successful culture usually results from the selection of a unique cultivatable clone, the quantity of inoculated pathogen should be as high as possible. Samples should be collected from anatomic sites that are likely to contain a high concentration of bacteria, and inoculation of a large volume of tissue sample is preferable. In patients with *Bartonella* endocarditis, the sensitivity of cell cultures has been shown to be higher when performed with valvular biopsy samples than with peripheral blood samples (*49*). These criteria, however, are not always feasible, as patients may reject, for instance, invasive explorations that are required to obtain the specimens. Arthropod-transmitted bacteria, which are often rare in infected human tissues, may be sometimes more easily recovered from samples collected from infected vectors; this was the key leading to the identification of *B. burgdorferi* (*19*). If initially such a result was insufficient in a clinical diagnostic approach, it has since led to efficient serologic and molecular tools, which would not have been available without culture. For intracellular bacteria, the use of a lysis method for eukaryotic cells before inoculation substantially enhances the ability to grow the organisms, especially when inoculation is performed in an axenic media, as for *Bartonella* or *Mycobacterium* species (*15*). Since low-speed centrifugation may also increase infectivity, the centrifugation-shell vial technique for isolating cytomegalovirus has been adapted to detect intracellular bacteria and used successfully to cultivate *Rickettsia* species from blood and skin biopsies and *T. whipplei* from the mitral valve of a patient with endocarditis (*2*).

**Table 2:** Key issues for isolating main emerging bacteria

	Medium			Conditions for incubation		
Group	Axenic specific medium	Living system (embryonated egg, cell line)		Low temperature (<37°C)	O2 and CO2 conditions	Extended incubation	
						
Alpha1 Proteo- bacteriae		*Ehrlichia* sp. *Rickettsia* sp. *Chlamydia* sp.		ELB agent (“*Rickettsia felis*”) (28°C)		*Ehrlichia* sp. *Rickettsia* sp.	
Alpha2 Proteo- bacteriae	*Afipia* sp.	*Afipia* sp. *Bartonella* sp.		*Bartonella bacilliformis* (28°C)		*Bartonella* sp.	
Spirochetae	*Borrelia* sp.			*Treponema pallidum*			
Delta-Xi Proteo- bacteriae					*Campylobacter* sp. (microaerophilic) *Helicobacter* sp. (microaerophilic)	*Helicobacter pylori*	
Gamma Proteo- bacteriae	*Legionella* sp.	*Legionella* sp.		*Yersinia pestis*			
Mycobacteria	*Mycobacterium* sp.			*Mycobacterium leprae*	*Mycobacterium malmoense *(microaerophilic)	*Mycobacterium* sp.	
Mycoplasmas	*Mycoplasma* sp.					*Mycoplasma fermentans*	
Gram-positive bacteria		*Tropheryma whipplei*			*Clostridium difficile* (anaerobic)	*Tropheryma whipplei*	

Special attention should be accorded to the duration, temperature, and atmosphere of incubation. For some of the most important newly discovered pathogens, such as *H. pylori*, patience has been a key to successful cultivation (*25*). With *T. whipplei*, the first evidence of cytopathic effect and microorganisms did not occur until day 65 after inoculation (*2*). Isolation of *Bartonella henselae* from blood or tissue samples from infected patients required up to 33 days’ incubation (*15*,*49*). Although most pathogenic bacteria have been cultured at 35°C to 37°C, which is close to the physiologic temperature of the human body, several pathogens need a lower temperature. In addition to well-known examples such as *M. leprae* and *Treponema pallidum***,** several arthropod-borne pathogens, including arboviruses, *Yersinia pestis*, *B. bacilliformis*, or *R. felis* may be more easily cultivated at <32°C (*8*).

## A More Recent Technique: 16S rDNA Amplification and Sequencing

With the use of universal primers that recognize highly conservative loci such as the 16S rDNA encoding gene, species-specific sequences can be amplified directly from diseased host tissues and compared with a reference-sequence database to infer phylogenetic relationships (*3*,*4*). This broad-range PCR technique has expanded the ability of laboratories to partially characterize organisms that had never been cultured. Thus, in the last decade, it has enabled two unexplained illnesses to be associated with novel etiologic agents: *B. henselae* in bacillary angiomatosis and 1 year later *T. whipplei* in patients with Whipple disease (*52*,*53*). These remarkable successes of molecular techniques, however, should not obscure the fact that a bacterial origin was previously established for both diseases on the basis of histologic studies and clinical responses to antimicrobial treatment (*2*,*48*). Further, isolation and culture were achieved at the same time as molecular identification (for *B. henselae*) or soon after (*for T. whipplei*) (*2*,*15*). In both cases, successful isolation resulted from laboratory practices generally used to enhance the detection of fastidious pathogens. Although it has been suggested that specific culture conditions could be inferred from molecular phylogenetic data, such a situation has never occurred for any bacterium (*3*). These examples suggest, therefore, that molecular techniques are particularly useful for taxonomic studies and identification, while traditional methods remain powerful to detect pathogens.

For viruses, several species, such as the *Sin Nombre virus* (SNV) or the *Hepatitis C virus* (HCV*)*, were detected by reverse transcriptase PCR before any morphologic, serologic, or cultural detection. Although SNV was subsequently cultured in vitro, the HCV agent has only been cultured recently in chimeric mice (*54*). Because of its high sensitivity, broad-range PCR also expands the ability to detect organisms present in very low quantity and those that are difficult to grow, such as intracellular bacteria. *Ehrlichia ewingii*, previously known as a canine parasite, was detected by this technique in circulating leukocytes of four patients with febrile illness. Note, however, that morulae had been identified in neutrophils from two of the four patients, providing strong evidence for an ehrlichial origin for the disease, and that serologic evidence was reported before the PCR assays (*6*). Advantages of molecular techniques seem more obvious for *Bartonella grahamii* and *B. vinsonii* subsp*. berkhoffii*, which have been implicated in human disease solely on the basis of 16S rDNA amplification and sequencing (*14*,*55*). Molecular tools are also particularly useful in diseases associated with dormant or latent organisms, such as chronic Lyme arthritis, and for which the sensitivity of culture from body fluids remains very low (*4*,). The advantages of broad-range PCR, however, are offset by the problem of microbial DNA contamination. Even after rigorous technical precautions are taken to minimize contamination of PCR reaction, false-positive reactions can occur. Another noticeable limitation of broad-range PCR is the examination of sites that are not normally sterile, such as feces or sputum; use of family-restricted primers, in situ hybridization with specific nucleic probes, or expression library screening with immune sera may help to evercome such limitations (*3*,*4*). Another potential problem is interpretation of the microheterogeneity found in microbial sequences derived directly from host tissues, especially when these sequences become the sole basis for defining the existence of an organism. For example, attempts to characterize and classify nanobacteria using 16S rDNA sequence analysis provided doubtful results, and these organisms were later considered contamination (*56*). Additionally, current databases contain an insufficient number of entries with which to define species and other taxon boundaries over a wide range of microorganisms (*3*).

## Advantages of Culture for the Study of Emerging Bacterial Diseases

### Antibiotic Susceptibility Testing

When culture and isolation are achieved, susceptibility of emerging bacteria to a large panel of antimicrobial drugs can be easily tested, providing essential data to guide clinical treatment, particularly when resistant strains are reported and empiric therapy may be ineffective. This antimicrobial testing would have been difficult, if not impossible, with molecular techniques, as genetic determinants of antibiotic resistance have been identified in only a few situations (*57*). Thus, isolation of *H. pylori* has revolutionized the treatment of duodenal ulcers, which are now definitively healed by appropriate antimicrobial regimens. As strains resistant to either metronidazole or clarithromycin have been increasingly reported, culture of the agent is very helpful in case of proven treatment failure, to assess the antibiotic resistance pattern of local strains of *H. pylori* (*58*). Coculture of bacteria with cell lines has brought new insights about antibiotic susceptibility patterns for obligate and facultative intracellular organisms. For example, while patients with human ehrlichiosis have been treated for a long time, with variable results, with chloramphenicol, in vitro studies showed that *E. chaffeensis* was resistant to this antibiotic (*59*).

## Experimental Animal Models for Pathogenicity

With viable microorganisms, disease models can often be established in animals. Rodent models are the most commonly used. For *Legionella oakridgensis*, originally isolated from industrial cooling towers, demonstration of its pathogenicity for guinea pigs suggested for the first time, before any clinical involvement, that it might be an unrecognized human pathogen (*29*). For assessing the capability of various *Vibrio* species to elaborate an enterotoxin, rabbit and mouse intestinal models were used (*33*). Human tissues can also now be maintained in immunodeficient mice (SCID-hu), which can then serve as useful models for human host-specific pathogens (*56*,*60*). Although less accessible, primate models supported, for example, the implication of *Mycoplasma genitalium* in genital tract infections (*35*). Finally, experimental animal models are useful for immunization studies, as for *H. pylori* in mouse and primate models. Following culture, immunodominant antigens can be cloned, expressed, and inoculated to animals to identify candidate vaccines (*61*).

### Genetic Studies

#### Isolated Genes

For noncultured organisms, molecular techniques have been proposed to identify isolated bacterial genes directly from clinical specimens. These techniques, however, are quite difficult to use and can identify only a few, short genetic fragments (*3*). On the other hand, by providing pure microbial cell mass, culture enables genes to be identified in high numbers through recombinant chromosomal libraries built from the extracted DNA. Genes identified in this fashion can then be utilized as more refined diagnostic tools. For example, *Rickettsia mongolotimonae* and *R. slovaca* were associated with human disease on the basis of amplification of a species-specific *rOmpA* gene fragment from skin biopsy specimens (*11*,*12*). DNA probes developed after isolation of *Chlamydia pneumoniae* enabled this organism to be detected by in situ hybridization in coronary atherosclerotic plaques (*62*). Further, molecular subtyping of cultured strains has offered new perspectives for epidemiologic studies. Thus, comparison of nucleotide sequences of 16S rDNA, *OspA,* and *Fla* genes for different strains of *B. burgdorferi* provided phylogenetic data that consistently supported the division of *B. burgdorferi* sensu lato into three geographically distinct genotypes, which were subsequently shown to have different pathogenic potentials (*63*). Correlation between genotypes and biologic characters is a key to understanding the pathophysiology of bacterial diseases.

#### Complete Genome Sequence

Because of the importance of organisms such as *H. pylori, M. genitalium,* and *C. pneumoniae* as emerging human pathogens and the value of complete genome sequence information for drug discovery and vaccine development, the complete nucleotide sequences of these three organisms has been determined by the whole-genome random sequencing method as described initially for *Haemophilus influenzae*. Sequence analyses allowed identification of several predicted coding regions that included genes required for DNA replication, transcription and translation, DNA repair, cellular transport, and energy metabolism (*64*). With the availability of complete genome sequences, further assessment of microbial genetic diversity is possible; based on the large number of sequence-related genes encoding outer membrane proteins, *H. pylori* was predicted to use recombination as a mechanism for antigenic variation and adaptative evolution (*65*). As the genome sequences of new bacterial species or strains are determined, comparative genomics will be an increasingly useful method to provide insights into physiologic differences among microorganisms (*64*).

## Conclusion

A comprehensive study of the histories of emerging bacterial diseases provided new insights into the respective roles played by the different identification techniques. Because of the spectacular development of molecular methods, traditional techniques have been prematurely considered obsolescent. We hope to have shown, however, that such a statement does not reflect the real contribution of these techniques. The undoubted value of novel molecular methods, especially for rapid bacterial detection and phylogenetic studies, should not hide the crucial role that traditional techniques have historically played. Moreover, these traditional techniques have never stopped evolving towards increased sensitivity and specificity. Today, these techniques appear complementary. If broad-range PCR was helpful in determining the taxonomic position of new, still uncultured organisms, most of the novel infectious diseases were finally described after culture and isolation of the responsible agents. In the current, fast-changing world of emerging infections, fulfillment of Koch’s postulates, which requires culture, remains a very necessary model of rigorous proof and scientific thinking (*1*). Culture is still an irreplaceable key for studying emerging bacterial diseases, even if routine diagnosis can be efficiently achieved by using other (although generally culture-derived) tools, including genetic amplification. The history of infectious diseases shows that no human bacterial pathogen is uncultivable so far: the real issue seems to be whether we are able to determine the environmental conditions required by prokaryotic agents for growth (*2*).

## References

[1] Krause RM. Microbes and emerging infections: the compulsion to become something new. ASM News. 2001;65:15–20.

[2] Raoult D, Birg ML, La Scola B, Fournier PE, Enea M, Lepidi H, Cultivation of the bacillus of Whipple disease. N Engl J Med. 2000;342:620–5. 10.1056/NEJM200003023420903 10699161

[3] Relman DA. The search for unrecognized pathogens. Science. 1999;284:1308–10. 10.1126/science.284.5418.1308 10334977

[4] Fredricks DN, Relman DA. Sequence-based identification of microbial pathogens: a reconsideration of Koch’s postulates. Clin Microbiol Rev. 1996;9:18–33. 8665474 10.1128/cmr.9.1.18 PMC172879

[5] Maeda K, Markowitz N, Hawley RC, Ristic M, Cox D, McDade JE. Human infection with *Ehrlichia canis*, a leukocytic rickettsia. N Engl J Med. 1987;316:853–6. 3029590 10.1056/NEJM198704023161406

[6] Buller RS, Arens M, Hmiel SP, Paddock CD, Sumner JW, Rikhisa Y, *Ehrlichia ewingii*, a newly recognized agent of human ehrlichiosis. N Engl J Med. 1999;341:148–55. 10.1056/NEJM199907153410303 10403852

[7] Goodman JL, Nelson C, Vitale B, Madigan JE, Dumler JS, Kurtti TJ, Direct cultivation of the causative agent of human granulocytic ehrlichiosis. N Engl J Med. 1996;334:209–15. 10.1056/NEJM199601253340401 8531996

[8] Raoult D, La Scola B, Enea M, Fournier PE, Roux V, Fenollar F, Isolation and characterization of a flea-associated rickettsia pathogenic for humans. Emerg Infect Dis. 2001;7:73–81. 11266297 10.3201/eid0701.010112 PMC2631683

[9] Uchida T. *Rickettsia japonica*, the etiologic agent of oriental spotted fever. Microbiol Immunol. 1993;37:91–102. 8502180 10.1111/j.1348-0421.1993.tb03185.x

[10] Raoult D, Brouqui P, Roux V. A new spotted fever group Rickettsiosis. Lancet. 1996;348:412. 10.1016/S0140-6736(05)65037-4 8709763

[11] Raoult D, Berbis P, Roux V, Xu W, Maurin M. A new tick-transmitted disease due to *Rickettsia slovaca.* Lancet. 1997;350:112–3. 10.1016/S0140-6736(05)61814-4 9228967

[12] Brenner DJ, Hollis DG, Moss CW, English CK, Hall GS, Vincent J, Proposal of *Afipia* gen. nov., with *Afipia felis* sp. nov. (formerly the cat scratch disease bacillus), *Afipia clevelandensis* sp. nov. (formerly the Cleveland Clinic Foundation strain), *Afipia broomeae* sp. nov., and three unnamed genospecies. J Clin Microbiol. 1991;29:2450–60. 1774249 10.1128/jcm.29.11.2450-2460.1991 PMC270354

[13] Daly JS, Worthington MG, Brenner DJ, Moss WC, Hollis DG, Weyant RS, *Rochalimaea elizabethae* sp. nov. isolated from a patient with endocarditis. J Clin Microbiol. 1993;31:872–81. 7681847 10.1128/jcm.31.4.872-881.1993 PMC263580

[14] Kerkhoff FT, Bergmans AMC, van der Zee A, Rothova A. Demonstration of *Bartonella grahamii* DNA in ocular fluids of a patient with neuroretinitis. J Clin Microbiol. 1999;37:4034–8. 10565926 10.1128/jcm.37.12.4034-4038.1999 PMC85873

[15] Slater LN, Welch DF, Hensel D, Coody DW. A newly recognized fastidious gram-negative pathogen as a cause of fever and bacteremia. N Engl J Med. 1990;323:1587–93. 2233947 10.1056/NEJM199012063232303

[16] Regnery RL, Olson JG, Perkins BA, Bibb W. Serological response to *Rochalimaea henselae* antigen in suspected cat-scratch disease. Lancet. 1992;339:1443–5. 10.1016/0140-6736(92)92032-B 1351130

[17] Vandamme P, Heyndrickx M, Vancanneyt M, Hoste B, de Vos P, Falsen E, *Bordetella trematum* sp. nov., isolated from wounds and ear infections in humans, and reassessment of *Alcaligenes denitrificans* Rüger and Tan 1983. Int J Syst Bacteriol. 1996;46:849–85. 8863408 10.1099/00207713-46-4-849

[18] Homes B, Costas M, On SL, Vandamme P, Falsen E, Kersters K. *Neisseria weaveri* sp. nov. (formerly CDC group M-5), from dog bite wounds of humans. Int J Syst Bacteriol. 1993;43:693. 10.1099/00207713-43-4-687 8240951

[19] Burgdorfer W. Discovery of the Lyme disease spirochete and its relation to tick vector. Yale J Biol Med. 1984;57:515–20. 6516454 PMC2590008

[20] Cutler SJ, Fekade D, Hussein K, Knox KA, Melka A, Cann K, Successful in-vitro cultivation of *Borrelia recurrentis.* Lancet. 1994;343:242. 10.1016/S0140-6736(94)91032-4 7904703

[21] Cutler SJ, Moss J, Fukunaga M, Wright DJM, Fekade D, Warrell D. *Borrelia recurrentis* characterization and comparison with relapsing-fever, lyme-associated, and other Borrelia spp. Int J Syst Bacteriol. 1997;47:958–68. 9336893 10.1099/00207713-47-4-958

[22] Taylor DN, Kiehlbauch JA, Tee W, Pitarangsi C, Echeverria P. Isolation of group 2 aerotolerant *Campylobacter* species from Thai children with diarrhea. J Infect Dis. 1991;163:1062–7. 2019754 10.1093/infdis/163.5.1062

[23] Totten PA, Fennell CL, Tenover FC, Wezenberg JM, Perine PL, Stamm WE, *Campylobacter cinaedi* (sp. nov.) and *Campylobacter Campylobacter* (sp. nov.): two new *Campylobacter* species associated with enteric disease in homosexual men. J Infect Dis. 1985;151:131–9. 3965584 10.1093/infdis/151.1.131

[24] Heilmann KL, Borchard F. Gastritis due to spiral shaped bacteria other than *Helicobacter pylori*: clinical, histological, and ultrastructural findings. Gut. 1991;32:137–40. 10.1136/gut.32.2.137 1864530 PMC1378794

[25] Marshall B. Unidentified curved bacillis on gastric epithélium in active chronic gastritis. Lancet. 1983;I:1273–5. 6134060

[26] Tarr PI, Fouser LS, Stapleton AE, Wilson RA, Kim HH, Vary JC, Hemolytic uremic syndrome in a six-year-old girl after a urinary tract infection with Shiga-toxin-producing *Escherichia coli* O103:H2. N Engl J Med. 1996;335:635–8. 10.1056/NEJM199608293350905 8687518

[27] The Brazilian Purpuric Fever Study Group. *Haemophilus aegyptus* bacteremia in Brazilian purpuric fever. Lancet. 1987;331:761–3. 2888986

[28] Fernstersheib MD, Miller M, Diggins C, Liska S, Detwiler L, Werner SB, Outbreak of Pontiac fever due to *Legionella anisa.* Lancet. 1990;336:35–7. 10.1016/0140-6736(90)91532-F 1973219

[29] Korvick JA, Yu VL, Fang GD. *Legionella* species as hospital-acquired respiratory pathogens. Semin Respir Infect. 1987;2:34–47. 3321265

[30] Herwaldt LA, Gorman GW, McGrath T, Toma S, Brake B, Hightower AW, A new *Legionella* species, *Legionella feeleii* species nova, causes Pontiac fever in an automobile plant. Ann Intern Med. 1984;100:333–8. 6696354 10.7326/0003-4819-100-3-333

[31] McDade JE, Brenner DJ, Bozeman M. Legionnaires’ disease bacterium isolated in 1947. Ann Intern Med. 1979;90:659–61. 373548 10.7326/0003-4819-90-4-659

[32] Birtles RJ, Rowbotham TJ, Raoult D, Harrison TG. Phylogenetic diversity of intra-amoebal legionellae as revealed by 16S rRNA gene sequence comparison. Microbiology. 1996;142:3525–30. 9004515 10.1099/13500872-142-12-3525

[33] Janda JM, Powers C, Bryant RG, Abbott SL. Current perspectives on the epidemiology and pathogenesis of clinically significant *Vibrio* spp. Clin Microbiol Rev. 1988;1:245–67. 3058295 10.1128/cmr.1.3.245 PMC358049

[34] Falkinham JO. Epidemiology of infection by nontuberculous mycobacteria. Clin Microbiol Rev. 1996;9:177–215. 8964035 10.1128/cmr.9.2.177 PMC172890

[35] Taylor-Robinson D. Infections due to species of *Mycoplasma* and *Ureaplasma*: an update. [quiz 683-4]. Clin Infect Dis. 1996;23:671–82. 8909826 10.1093/clinids/23.4.671

[36] La Scola B, Fenollar F, Fournier PE, Altwegg M, Mallet MN, Raoult D. Description of *Tropheryma whipplei* gen. nov. sp. nov., the Whipple disease bacillus. Int J Syst Bacteriol. 2001;51:1471–9. 10.1099/00207713-51-4-1471 11491348

[37] Funke G, Lawson PA, Collins MD. Heterogeneity within human-derived centers for disease control and prevention (CDC) coryneform group ANF-1-like bacteria and description of *Corynebacterium auris* sp. nov. Int J Syst Bacteriol. 1995;45:735–9. 7547292 10.1099/00207713-45-4-735

[38] Freney J, Brun Y, Bes M, Meugnier H, Grimont F, Grimont PAD, *Staphylococcus lugdunensis* sp. nov. and *Staphylococcus schleiferi* sp. nov., two species from human clinical specimens. Int J Syst Bacteriol. 1988;38:168–72.

[39] Centers for Disease Control and Prevention. Invasive infection with *Streptococcus iniae*--Ontario, 1995-1996. MMWR Morb Mortal Wkly Rep. 1996;45:650–3. 8769472

[40] Woods GL, Walker DH. Detection of infection or infectious agents by use of cytologic and histologic stains. Clin Microbiol Rev. 1996;9:382–404. 8809467 10.1128/cmr.9.3.382 PMC172900

[41] Maurin M, Raoult D. *Bartonella* (*Rochalimaea*) *quintana* infections. Clin Microbiol Rev. 1996;9:273–92. 8809460 10.1128/cmr.9.3.273 PMC172893

[42] Curry A. Electron microscopy as a tool for identifying new pathogens. J Infect. 2000;40:107–15. 10.1053/jinf.2000.0634 10841083

[43] La Scola B, Raoult D. *Afipia felis* in a hospital water supply in association with free-living amoebae. Lancet. 1999;353:1330. 10.1016/S0140-6736(99)00906-X 10218538

[44] Sovilla JY, Regli F, Francioli PB. Guillain-Barré syndrome following *Campylobacter jejuni* enteritis: report of three cases and review of the literature. Arch Intern Med. 1988;148:739–41. 10.1001/archinte.148.3.739 3277576

[45] Tarasevich IV, Makarova VA, Fetisova NF, Stepanov AV, Miskarova ED, Balayeva N, Astrakhan fever, a spotted-fever rickettsiosis. Lancet. 1991;337:172–3. 10.1016/0140-6736(91)90833-B 1670806

[46] Dumler J, Walker D. Tick-borne ehrlichioses. Lancet Infect Dis. 2001;:21–8.11871406

[47] La Scola B, Michel G, Raoult D. Isolation of *Legionella pneumophila* by centrifugation of shell vial cell cultures from multiple liver and lung abscesses. J Clin Microbiol. 1999;37:785–7. 9986854 10.1128/jcm.37.3.785-787.1999 PMC84555

[48] Koehler JE, Quinn FD, Berger TG, Leboit PE, Tappero JW. Isolation of *Rochalimaea* species from cutaneous and osseous lesions of bacillary angiomatosis. N Engl J Med. 1992;327:1625–31. 1435899 10.1056/NEJM199212033272303

[49] La Scola B, Raoult D. Culture of *Bartonella quintana* and *Bartonella henselae* from human samples: a 5-year experience (1993 to 1998). J Clin Microbiol. 1999;37:1899–905. 10325344 10.1128/jcm.37.6.1899-1905.1999 PMC84980

[50] Knoop FC, Owens M, Crocker IC. *Clostridium difficile*: clinical disease and diagnosis. Clin Microbiol Rev. 1993;6:251–65. 8358706 10.1128/cmr.6.3.251 PMC358285

[51] Birtles RJ, Rowbotham TJ, Storey C, Marrie TJ, Raoult D. Chlamydia-like obligate parasite of free-living amoebae. Lancet. 1997;349:925–6. 10.1016/S0140-6736(05)62701-8 9093261

[52] Relman AD, Schmidt TM, MacDermott RP, Falkow S. Identification of the uncultured bacillus of Whipple disease. N Engl J Med. 1992;327:293–301. 1377787 10.1056/nejm199207303270501 PMC13545976

[53] Relman DA, Loutit JS, Schmidt TM, Falkow S, Tompkins LS. The agent of bacillary angiomatosis. An approach to the identification of uncultured pathogens. N Engl J Med. 1990;323:1573–80. 2233945 10.1056/nejm199012063232301 PMC13632802

[54] Mercer DF, Schiller DE, Elliott JF, Douglas DN, Hao C, Rinfret A, Hepatitis C virus replication in mice with chimeric human liers. Nat Med. 2001;7:927–33. 10.1038/90968 11479625

[55] Roux V, Eykyn SJ, Wyllie S, Raoult D. *Bartonella vinsonii* subsp. *berkhoffii* as an agent of afebrile blood culture-negative endocarditis in a human. J Clin Microbiol. 2000;38:1698–700. 10747175 10.1128/jcm.38.4.1698-1700.2000 PMC86533

[56] Cisar JO, Thompson J, Swain W, Hu L, Kopecko D. An alternative interpretation of nanobacteria induced biomineralization. Proc Natl Acad Sci U S A. 2000;97:11511–5. 10.1073/pnas.97.21.11511 11027350 PMC17231

[57] Drancourt M, Raoult D. Characterization of mutations in the *rpoB* gene in naturally rifampin-resistant *Rickettsia* species. Antimicrob Agents Chemother. 1999;43:2400–3. 10508014 10.1128/aac.43.10.2400 PMC89490

[58] Goodwin CS, Mendall MM, Northfield TC. *Helicobacter pylori* infection. Lancet. 1997;349:265–9. 10.1016/S0140-6736(96)07023-7 9014926

[59] Brouqui P, Raoult D. In vitro antibiotic susceptibility of the newly recognized agent of ehrlichiosis in humans, *Ehrlichia chaffeensis.* Antimicrob Agents Chemother. 1992;36:2799–803. 1482148 10.1128/aac.36.12.2799 PMC245548

[60] MacCune JM, Namikawa R, Kaneshima H, Shultz LD, Lieberman M, Weissman IL. The SCID-hu mouse: murine model for the analysis of human hematolymphoid differentiation and function. Science. 1988;241:1632–9. 10.1126/science.2971269 2971269

[61] Sutton P, Lee A. Review article: *Helicobacter pylori* vaccines – the current status. Aliment Pharmacol Ther. 2000;14:1107–18. 10.1046/j.1365-2036.2000.00825.x 10971226

[62] Kuo CC, Jackson LA, Campbell LA, Grayston JT. *Chlamydia pneumoniae* (TWAR). Clin Microbiol Rev. 1995;8:451–61. 8665464 10.1128/cmr.8.4.451 PMC172870

[63] Balmelli T, Piffaretti JC. Association between different clinical manifestations of Lyme disease and different species of *Borrelia burgdorferi* sensu lato. Res Microbiol. 1995;146:329–40. 10.1016/0923-2508(96)81056-4 7569327

[64] Read TD, Brunham RC, Shen C, Gill SR, Heidelberg JF, White O, Genome sequences of *Chlamydia trachomatis* MoPn and *Chlamydia pneumoniae* AR39. Nucleic Acids Res. 2000;28:1397–406. 10.1093/nar/28.6.1397 10684935 PMC111046

[65] Tomb JF, White O, Kerlavage AR, Clayton RA, Sutton GG, Fleishmann RD, The complete genome sequence of the gastric pathogen *Helicobacter pylori.* Nature. 1997;388:539–47. 10.1038/41483 9252185

